# Pathway-level mutation analysis in primary high-grade serous ovarian cancer and matched brain metastases

**DOI:** 10.1038/s41598-022-23788-4

**Published:** 2022-11-29

**Authors:** Renata Duchnowska, Anna Maria Supernat, Rafał Pęksa, Marta Łukasiewicz, Tomasz Stokowy, Roy Ronen, Janusz Dutkowski, Monika Umińska, Ewa Iżycka-Świeszewska, Anna Kowalczyk, Waldemar Och, Monika Rucińska, Wojciech P. Olszewski, Tomasz Mandat, Bożena Jarosz, Michał Bieńkowski, Wojciech Biernat, Jacek Jassem

**Affiliations:** 1 Oncology Department, Military Institute of Medicine - National Research Institute, Szaserów St. 128, 04-141 Warsaw, Poland; 2 Laboratory of Translational Oncology, Intercollegiate Faculty of Biotechnology, University of Gdańsk and Medical University of Gdańsk, Gdańsk, Poland; 3 Department of Pathology, Medical University of Gdańsk, Gdańsk, Poland; 4 Department of Clinical Science, University of Bergen, Bergen, Norway; 5 Data4Cure, Inc., La Jolla, CA USA; 6 Oncology Department Oncology Center, Gdynia, Poland; 7 Department of Pathology and Neuropathology, Medical University of Gdańsk, Gdańsk, Poland; 8 Department of Oncology and Radiotherapy, Medical University of Gdańsk, Gdańsk, Poland; 9 Neurosurgery Department, Regional Specialist Hospital, Olsztyn, Poland; 10 Department of Oncology, Collegium Medicum, University of Warmia and Mazury, Olsztyn, Poland; 11 Department of Pathology, Maria Skłodowska-Curie Memorial Cancer Center and Institute of Oncology, Warsaw, Poland; 12 Department of Neurosurgery, Maria Skłodowska-Curie Memorial Cancer Center and Institute of Oncology, Warsaw, Poland; 13 Department of Neurosurgery and Paediatric Neurosurgery, Medical University of Lublin, Lublin, Poland

**Keywords:** Cancer, Molecular biology, Neuroscience, Diseases, Medical research, Molecular medicine, Oncology, Pathogenesis

## Abstract

Brain metastases (BMs) in ovarian cancer (OC) are a rare event. BMs occur most frequently in high-grade serous (HGS) OC. The molecular features of BMs in HGSOC are poorly understood. We performed a whole-exome sequencing analysis of ten matched pairs of formalin-fixed paraffin-embedded samples from primary HGSOC and corresponding BMs. Enrichment significance (*p* value; false discovery rate) was computed using the Reactome, the Kyoto Encyclopedia of Genes and Genomes pathway collections, and the Gene Ontology Biological Processes. Germline DNA damage repair variants were found in seven cases (70%) and involved the *BRCA1*, *BRCA2*, *ATM*, *RAD50*, *ERCC4*, *RPA1*, *MLHI*, and *ATR* genes. Somatic mutations of *TP53* were found in nine cases (90%) and were the only stable mutations between the primary tumor and BMs. Disturbed pathways in BMs versus primary HGSOC constituted a complex network and included the cell cycle, the degradation of the extracellular matrix, cell junction organization, nucleotide metabolism, lipid metabolism, the immune system, G-protein-coupled receptors, intracellular vesicular transport, and reaction to chemical stimuli (Golgi vesicle transport and olfactory signaling). Pathway analysis approaches allow for a more intuitive interpretation of the data as compared to considering single-gene aberrations and provide an opportunity to identify clinically informative alterations in HGSOC BM.

## Introduction

Ovarian cancer (OC) is the eighth most common malignancy and the eighth cause of cancer death in females globally^1^. The majority of OC patients present in clinical stages III and IV^2^. Brain metastases (BMs) in OC are a rare event, with an estimated risk of 1.3%^3^. BMs in OC usually originate from the high-grade serous histology type (HGSOC), constituting approximately 75% of all OCs^3,4^. The prognosis of OC in patients with BMs is poor, with a median overall survival of around ten months^3,4^. The mutation spectrum in HGSOC is different than in other subtypes of OC. Significantly mutated genes in HGSOC include *TP53, BRCA1*, *CSMD3*, *NF1*, *CDK12*, *FAT3*, *GABRA6*, *BRCA2*, and *RB1*^5,6^. Interestingly, the HGSOC mutation spectrum is reminiscent of that observed in basal-like breast cancers, which are characterized by a particularly high risk of BM^7^. A few studies have reported an increased risk of BMs in OC patients with germline *BRCA1* and *BRCA2* mutations and androgen receptor expression in the primary tumor^8–10^. The molecular features of BMs in OC are poorly understood. In prior studies, the most common alterations were found in the *BRCA1/2*, *MDR1*, *IL7R*, *CALB2*, *CYP1B1*, *EFTUD1*, *RARRES2*, *TIMP3*, *MYC*, I*RF1*, *BCL2L2*, and *TNFSF10* genes, as well as in the WNT-b-catenin, JAK-STAT, and NOTCH pathways^8–16^. The present study characterized genetic and pathway-level alterations in a unique series of primary HGSOC and matched BMs to identify abnormalities of potential clinical relevance.

## Results

The initial study group included 13 OC patients staged II-IV (International Federation of Gynecology and Obstetrics classification). All identified cases were ultimately found to be HGSOC. Subsequently, three patients were excluded due to insufficient tissue quality. The mean age of patients at primary OC diagnosis was 48 years (35–59 years; Table 1). All patients after surgery received one to seven lines of chemotherapy (median one) in the adjuvant or palliative setting. The median time from primary OC diagnosis to BM occurrence was 38 months (interquartile range; IQR, from 24 to 72 months). The median overall survival from primary OC diagnosis was 97 months (IQR, from 75 to 150 months), and the median overall survival from BM occurrence was 31 months (IQR, from 14 to 69 months).

**Table 1:** Patient characteristics (n = 10).

Variable	n (%)
**Age at primary tumor diagnosis (years)**	
Mean	48
Range	35–59
**Age at brain metastasis diagnosis (years)**	
Mean	55
Range	38–64
**Histology of primary ovarian tumor**	
High-grade serous epithelial carcinoma	10 (100)
**FIGO stage at diagnosis**	
II	1 (10)
III	6 (60)
IV	1 (10)
Unknown	2 (20)
**First line chemotherapy**	10 (100)
Paclitaxel + cisplatin	3 (30)
Paclitaxel + carboplatin	4 (40)
Unknown	3 (30)
**Number of chemotherapy lines before BM**	10 (100)
< 2 lines	4 (40)
≥ 2 lines	3 (30)
Unknown	3 (30)
**Extracranial disease**	10 (100)
Controlled	6 (60)
Uncontrolled	4 (40)
**Number of brain metastasis**	10 (100)
1	7 (70)
2–3	3 (30)
**Brain metastasis site**	10 (100)
Cerebellum	4 (40)
Parietal lobe	2 (20)
Temporal lobe	1 (10)
Occipital	1 (10)
Other	2 (20)

*FIGO* International Federation of Gynecology and Obstetrics classification, *BM* brain metastasis.

### DNA damage repair gene variants

Germline DNA damage repair (DDR) variants were found in the *BRCA1* gene (three cases), *BRCA2* (two), *ATM* (two), *RAD50* (four), *ERCC4* (one), *RPA1* (one), *MLH1* (one), and *ATR* (two cases). Four patients carried more than one germline mutation: *RAD50* and *ATR 13*; *BRCA1*, *ATM*, and *RAD50*; *BRCA2* and *RAD50*; and *BRCA1*, *RAD50*, *ERCC4*, *RPA1*, and *MLH1*. Nine patients (90%) carried *TP53* somatic mutations, including four missense, three stop-gain, one splice, and one frameshift mutation. The *TP53* mutations were consistent between the primary tumors and BMs (Table 2, Supplementary Fig. 1). In four patients, *TP53* mutation coincided with other somatic DDR alterations: *BRCA1, ATM,* and *MLH1; BRCA2*, *ATM*, and *ATR; BRCA1, BRCA2, ATM, ERCC4,* and *FANCD2;* and *BRCA2* and *ATR*. Somatic mutations found in the primary tumor included *BRCA1* (three cases), *BRCA2* (three), *ATM* (three), *ATR* (two), *ERCC4* (two), *FANCD2* (two), MLH1 (one), and *RAD50* (one case), Somatic DDR mutations were more common in primary tumors than in BMs (Table 2, Supplementary Fig. 1). Four carriers of germline mutations had additional somatic DDR mutations in the primary tumor: *ERCC4; BRCA1, ATM, FANCD2, and MLH1*; *BRCA1*; and *BRCA2*, *RAD50*, and *ATR* (Tables 2 Supplementary Fig. 1). Additionally, two carriers had somatic DDR mutations in BM: *ATM* and *BRCA1* (Table 2, Supplementary Fig. 1).

**Table 2:** Somatic mutations in primary ovarian tumor and brain metastasis: overview of *TP53* and selected DNA damage repair gene variants.

Gene	Patient	Chromosome	Start	Severity	Impact	Aa_change	Normal	Primary tumor	Brain metastasis
*TP53*	P2	chr17	7,577,586	HIGH	frameshift_variant	p.Ile232fs	T/T	T/TGGTGGTACAGTCAGAGCCAA	T/TGGTGGTACAGTCAGAGCCAA
	P3	chr17	7,577,531	MED	missense_variant	p.Pro250Leu	G/G	G/A	G/A
	P5	chr17	7,577,534	MED	missense_variant	p.Arg249Thr	C/C	C/G	C/G
	P6	chr17	7,574,002	HIGH	stop_gained	p.Arg342*	G/G	G/A	G/A
	P7	chr17	7,578,551	HIGH	stop_gained	p.Tyr126*	G/G	G/C	G/C
	P10	chr17	7,577,508	MED	missense_variant	p.Glu258Lys	C/C	C/T	C/T
	P11	chr17	7,574,033	HIGH	splice_acceptor_variant		C/C	C/T	C/T
	P12	chr17	7,578,262	HIGH	stop_gained	p.Arg196*	G/G	G/A	G/A
	P13	chr17	7,578,535	MED	missense_variant	p.Lys132Gln	T/T	T/G	T/G
*BRCA1*	P3	chr17	41,223,165	MED	missense_variant	p.Arg1610Cys	G/G	G/A	G/G
	P6	chr17	41,234,537	HIGH	frameshift_variant	p.Glu1413fs	GT/GT	GT/GT	GT/G
	P10	chr17	41,215,964	MED	missense_variant	p.Ala1714Val	G/G	G/G	G/A
		chr17	41,215,973	MED	splice_region_variant		G/G	G/G	G/A
*BRCA2*	P7	chr13	32,930,581	HIGH	stop_gained	p.Gln2485*	C/C	C/C	C/T
	P7	chr13	32,937,478	HIGH	stop_gained	p.Gln2714*	C/C	C/T	C/C
	P10	chr13	32,929,104	MED	missense_variant	p.Ser2372Leu	C/C	C/C	C/T
		chr13	32,929,191	MED	missense_variant	p.Arg2401Lys	G/G	G/G	G/A
		chr13	32,929,193	MED	missense_variant	p.Pro2402Ala	C/C	C/C	C/G
		chr13	32,953,589	MED	missense_variant	p.Arg2964Lys	G/G	G/G	G/A
	P13	chr13	32,929,307	MED	missense_variant	p.His2440Asn	C/C	C/A	C/C
*ATM*	P3	chr11	108,188,207	MED	missense_variant	p.Ala2103Thr	G/G	G/A	G/G
		chr11	108,199,756	MED	missense_variant	p.Val2367Leu	G/G	G/G	G/C
		chr11	108,201,089	MED	missense_variant	p.Arg2486Gln	G/G	G/A	G/G
	P7	chr11	108,196,194	MED	missense_variant	p.Arg2244Lys	G/G	G/A	G/G
	P10	chr11	108,122,685	MED	missense_variant	p.Met577Lys	TG/TG	TG/AA	TG/TG
*RAD50*	P13	chr5	131,925,533	MED	splice_region_variant		G/G	G/A	G/G
*ERCC4*	P1	chr16	14,020,484	HIGH	frameshift_variant	p.Arg153fs	TCG/TCG	TCG/T	TCG/TCG
	P1	chr16	14,020,479	MED	missense_variant	p.Leu151Phe	C/C	C/T	C/C
	P10	chr16	14,029,412	MED	missense_variant	p.Ala542Ser	G/G	G/G	G/T
*FANCD2*	P3	chr3	10,085,525	MED	missense_variant	p.Thr371Ile	C/C	C/T	C/C
		chr3	10,085,541	HIGH	frameshift_variant	p.His377fs	A/A	A/AAAGAACTTACAAGATCGGAGTTC	A/A
	P10	chr3	10,107,100	MED	missense_variant	p.Ala731Val	C/C	C/C	C/T
*MLH1*	P3	chr3	37,067,173	MED	missense_variant	p.Ser362Phe	C/C	C/T	C/C
*ATR*	P7	chr3	142,168,444	HIGH	splice_acceptor_variant		C/C	C/T	C/C
	P13	chr3	142,279,243	HIGH	stop_gained	p.Gln468*	G/G	G/A	G/G

P: patient; considering variants that are high-quality (Mutect2 filter PASS), impactful (SnpEff HIGH/MED), rare in germline populations (max-all-aaf < 0.001). Additional annotation includes previous observations in cancer (Cosmic, TCGA).

### Pathway-level mutation analysis

#### Primary tumor versus normal tissue

Pathway-level differences between primary OC and matched normal tissues were assessed using the Reactome (REACT) and the Kyoto Encyclopedia of Genes and Genomes (KEGG) pathway collections. In total, 22 and 44 pathways for somatic variants were enriched in OC using REACT and KEGG, respectively (FDR for both < 0.0001; Data not shown). Among the top significantly mutated pathways were the cell cycle checkpoint pathway, including somatic variants of *TP53, RAD9A, ATM, RFC4, ATR, PSMD3, ORC5, MCM6,* and *RPA1* (FDR = 2.98961e^-8^), and the p53 pathway, including somatic variants of *TP53, ATM, ZMAT3, ATR, IGFBP3,* and *THBS1* (FDR = 6.76136e^-8^). These pathways are linked to proliferation and tumor carcinogenesis.

#### Brain metastasis versus the primary tumor

**Figure 1:**
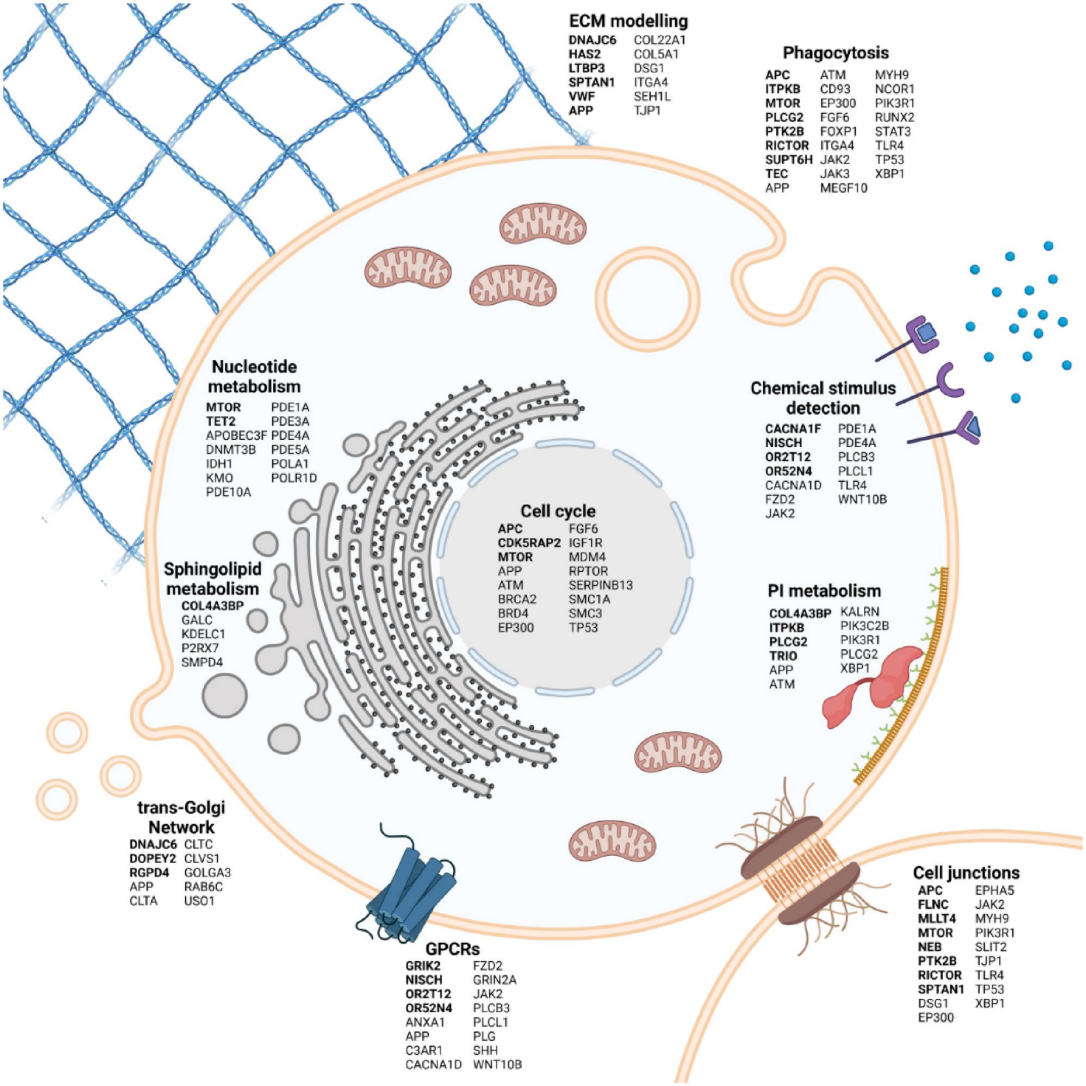
Main pathway altered in HGOC BMs (using REACT, KEGG and GO-BP pathway collections; genes altered in BM, in ≥ 2 patients are marked in bold. In this figure created in BioRender.com (https://biorender.com/) by Bieńkowski M., MD PhD with the assistance of The Excellence of Scientific Publications Unit, Medical University, Gdańsk, Poland.

A total of 33 REACT and 45 KEGG pathways, as well as 1,842 Gene Ontology Biological Processes (GO-BP) terms, were enriched for somatic variants in BMs (FDR < 0.01). Based on the previously stated assumptions, several pathway groups were found: the cell cycle (KEGG hsa04110; FDR = 0.008), ECM degradation (REACT_118572; FDR = 0.005) and cell junction organization (REACT_20676; FDR = 0.006), the disturbances of nucleotide metabolism (KEEOG, FDR = 0.003), phospholipid metabolism (REACT_120870; FDR = 0.005), toll-like receptor cascades (REACT_6966; FDR = 0.005), sphingolipid metabolism (REACT_19323; FDR < 0.01) and GPCR ligand binding (REACT_21340; FDR = 0.005), trans-Golgi network vesicle budding (REACT_11235; FDR = 0.005), clathrin-derived vesicle budding (REACT_19187; FDR = 0.005), taste transduction (KEEOG hsa04742; FDR = 0.003) and olfactory transduction (KEEOG hsa04740; FDR = 0.006), the sensory perception of chemical stimuli (GO: 0,007,606; FDR < 0.001), and G-protein-coupled receptor signaling pathway (GO: 0,007,186; FDR < 0.001) (Fig. 1, Supplementary Table 1).

## Discussion

This study provides a landscape of mutations and activated complex pathways in primary HGSOC and matched BM. Pathway analysis approaches lead to a more intuitive interpretation of the data than aberrations of single genes or proteins. Whereas single genes or proteins may only show non-significant changes, synchronous changes within a pathway may reveal a biologically significant effect. Hence, for some time, pathway analysis has become a crucial source for omics data analyses^17,18^. We found that the predominantly disturbed pathways in HGSOC BMs constitute a complex network and may be categorized into several groups, specifically those directly affecting tumor aggressiveness (the cell cycle, the degradation of the extracellular matrix, cell junction organization, and nucleotide metabolism), cellular membrane metabolism, the immune system (phospholipids, sphingolipids, toll-like receptors, and phagocytosis), processes related to intracellular vesicular transport and reaction to chemical stimuli (Golgi vesicle transport and olfactory signaling pathway), and connecting these pathways to G-protein-coupled receptors (GPCRs).

In our study, 80% of patients harbored germline DDR mutations, including four with more than one mutation. Previous studies reported an increased incidence of BMs in OC patients with germline mutations in *BRCA1* and *BRCA2* genes^8–10^. Also, HGSOC is associated with the near-universal presence of mutations in somatic *TP53* and genomic instability attributed to the absence of the fully functional repair of DNA double-strand breaks^6^. Indeed, in this series, 90% of cases carried somatic mutations in *TP53* that were consistent between primary tumor and BM. In turn, DDR somatic mutations were more common in the primary tumor than in BMs. Crucially, DDR is directly linked to innate immunity because cells are adept at sensing damaged and foreign DNA, and it often leads to increased mutational load and neoantigen burden. In our study, all BMs were metachronous, and the median time from primary OC diagnosis to BMs was 38 months. All patients underwent chemotherapy before the development of BMs, which may have induced some somatic alterations. Chemotherapy has previously been shown to alter the tumor immune profile and microenvironment, primarily through the induction of immunogenic cell death and the activation of interferon genes resulting in type I interferon production^19^. These and other signaling pathways may also result in the upregulation of PD-L1 expression, increased antigen presentation, and the expansion of neoantigen repertoires, all of which can increase clinical responses to immune-checkpoint blockade (ICB)^19,20^. This phenomenon is in line with the Darwinian model, in which therapeutic drugs or the tumor microenvironment act as a selective pressure by eliminating cellular clones with specific alterations and epigenetic or microenvironment features. This process may lead to the survival of the fittest clones and may contribute to clinical resistance to chemotherapy and targeted agents^21^. On the other hand, branched tumor evolution assumes that, although BMs and primary tumors share the same progenitor, they still diverge autonomously^22^.

The immune system seems to play a significant role in shaping the development of OC metastasis. Previous studies have shown that infiltrating regulatory T-cells and high programmed death-ligand 1 (PD-L1) expression in primary OC are associated with a metastatic phenotype and poor survival^23^. In contrast, the presence and extent of CD8 + tumor-infiltrating lymphocytes or an immunoreactive gene expression profile are associated with a favorable prognosis^24^. The immune system’s stimulation is also reflected in BMs from OC, i.e., by activating genes related to toll-like receptors expressed in neurons, astrocytes, and microglia. These receptors may mediate the link between inflammation and neurodegenerative diseases via the activation of brain microglia^25^. Hitherto, the immunological profile of BMs from OC has been examined in only two cases, and these lesions showed a high mutational burden and increased PD-L1 expression as compared with primary tumors^26^. The relationship between ICB response and DNA repair deserves special attention. Tumors with mismatch repair deficiency are highly responsive to ICB, and the FDA recently granted a breakthrough therapy designation to pembrolizumab to treat cancers with high microsatellite instability. However, the high mutational burden is neither necessary nor sufficient to drive ICB response, and distinct DNA lesions arising from different DNA repair-deficient backgrounds may produce different immunologic effects. A more thorough understanding of genomic instability—in all its forms—is essential for the potential clinical utility of this process. Monotherapy with immune checkpoint inhibitors induces response in 10–15% of heavily pre-treated OC patients^27,28^. Combined PD-L1 inhibitors and agents increasing tumor immunogenicity, such as poly ADP ribose polymerase inhibitors, are the subject of ongoing trials in OC.

Of particular note is the interaction between BMs and the ECM. The ECM is a highly dynamic structure and often undergoes remodeling in physiological and pathological processes. BMs disrupt the blood–brain barrier and create a complex multistep process in which the interaction with the ECM plays a crucial role in active extravasation, strict perivascular position, and cooptive growth^29^. In the current study, genes disturbed in ECM organization and disassembly included the cathepsin B (*CTSS*) and plasminogen genes. The *CTSS* gene plays a role in the polarization of antigen-presenting cells from the M1 toward the M2-phenotype and support for myeloid-derived suppressor cells and tumor-associated macrophages (TAM), whereas cysteine cathepsins are key regulators of the innate and adaptive arms of the immune system^30^. In a preclinical model, the inhibition of *CTSS* specifically impaired metastatic seeding and colonization in the brain^31^.

Interesting are also data concerning the plasminogen gene in the BM ECM. Tissue plasminogen activator (tPA) is present in the neurovascular unit cells and modulates its permeability in the hippocampus, hypothalamus, cerebellum, and amygdala^32^. Hence, tPA is an important element of the cross-talk between neurons and astrocytes^33^. The brain tPA/plasminogen cascade is highly dynamic, and its ability to assemble, disband, and reorganize is required to develop proper neuronal circuitry and facilitate post-injury repair processes^33^. In the past decade, several potent and selective compounds targeting the cancer cell-derived ECM and related enzymes have been developed^34^.

The brain is the tissue in humans with the second-highest lipid content and diversity in composition. There are three types of brain lipids: sphingolipids, glycerophospholipids, and cholesterol^35^. These lipids are components of the biological membranes, participate in cell signaling, and contribute to energy supply processes^35,36^. Disordered sphingolipid metabolism may lead to rearrangements in the formation of microdomains in the cell membrane and consequently affect synaptic transmission in neuronal-glial connections^37^. The blood–brain barrier is selectively disrupted in BM, partly via inhibiting endothelial cell-expressed docosahexaenoic acid (DHA) transporter, a significant facilitator of superfamily domain 2a (Mfsd2a). Mfsd2a expression is cooperatively regulated by the transforming growth factor β and basic fibroblast growth factor signaling pathways, which are pathologically diminished in the BM endothelium^38^. Disordered Mfsd2a is related to two other important pathways demonstrated in HGSOC BM: clathrin-mediated endocytosis and Golgi vesicle transport. Clathrin-mediated endocytosis, similar to caveolin-dependent endocytosis, participates in the uptake of extracellular vesicles (exosomes), that is, heterogeneous, membrane-bound packages containing complex cargo such as nucleic acids, lipids, and proteins. The exosomes secreted by cancer cells may transfer these proteins, mRNA, and miRNA; contribute to cell migration; and confer cytoprotective effects^39–41^. Interestingly, the loss of Mfsd2a may also increase caveolin-dependent endocytosis. Structural changes in and functional disorder of Golgi vesicle transport are involved in many human neurodegenerative diseases and cancers^42^. Most of these diseases are characterized by defects in membrane trafficking, such as the mislocalization of proteins, the impaired glycosylation of proteins, and the accumulation of undegraded proteins. Hence, extracellular vesicles seem to be an attractive diagnostic and therapeutic target. Similarly, restoring the DHA metabolism in the BM microenvironment may be viewed as a potential therapeutic strategy to block metastatic cell growth and survival. Finally, DHA obtained exclusively via dietary sources reduces neurocognitive deficits, e.g., in breast cancer patients receiving whole-brain irradiation, and improves response to chemotherapy^43^.

The role of GPCRs in BM is noteworthy. Specifically, GPCRs belong to the largest family of cell-surface molecules and are expressed in most cell types in the central and peripheral nervous tissues. They participate in vasculogenesis and angiogenesis in diverse tumor types; immune cell-mediated pathways; and proliferation, invasion, and survival at the secondary tumor site^44^. These GPCRs are stimulated by several ligands, chemokines, and accessory proteins, among which lysophosphatidic acid (LPA) and C-X-C motif chemokine receptor 4 deserve attention in OC. LPA is a bioactive lipid species, part of the lysophospholipid family, and involved in signal transduction between cells^45^. The level of LPA in plasma and ascites is increased even in the early stages of OC and has been considered a potential diagnostic marker in OC^46^. LPA stimulates the proliferation, migration, and invasion of OC cells through the regulation of several factors, i.e., vascular endothelial growth factor, tumor-necrosis factor α, and CXC chemokine ligand^47^. In the central nervous system, LPA signaling influences numerous processes, including the proliferation of neural precursor cells, neural and glial development, proper cell migration, and cell survival^48^. Environmental stressors, such as hypoxia, inflammation, and hemorrhage, which increase LPA signaling, are associated with multiple neuropathologies. Also, GPCRs are functionally linked to olfactory and taste transduction pathways^49,50^. The olfactory receptors (ORs) are frequently neglected in cancer genomic and transcriptomic studies because they are assumed to affect the olfactory epithelium exclusively^49^. However, recent reports indicate that some ORs display high expression in several cancer cell lines and may contribute to tumorigenesis^49^. The ability of ORs to sense small organic molecules may activate these pathways in cancer cells. On the other hand, this nose-brain pathway may allow for the rapid delivery of small therapeutic molecules to the brain, bypassing the blood–brain barrier. The manipulation of aberrant GPCR signaling is considered a potential therapeutic approach.

We are aware of several limitations of this study. First, we have not analyzed copy number variations (CNVs), which are common in genomically unstable HGSOC^6^. The prognostic value of the CNV-based risk scores in HGSOC seems to be promising but requires clinical validation^51^. Detecting CNV from exome sequencing is challenging due to the noncontiguous nature of the captured exons and the complex relationship between reading depth and copy number. Second, the homologous recombination deficiency (HRD) analysis used in our study included exclusively mutated genes. On the other hand, HR pathway gene mutations other than *BRCA1* and *BRCA2* are rare, and their association with HRD is unclear^6,52^. Further, a gene sequencing approach used in our study does not detect epigenetic events, such as *BRCA1* promoter methylation. Finally, we used formalin-fixed paraffin-embedded (FFPE) tissues, in which formaldehyde fixation may affect the DNA helix and lead to fixation artifacts. On the other hand, optimized DNA extraction and bioinformatics methods incorporating the genomic context allow for NGS studies in FFPE samples.

BMs still represent a challenging and unmet clinical problem. Therapeutic decisions in patients with BMs often consider only actionable alterations in the primary tumor. Our study provides the first data on the differences between functional pathways of primary HGSOC and metachronous BMs. Further investigations may optimize the management of HGSOC with BMs and pave the way for developing new preventive and therapeutic approaches.

## Materials and methods

### Patients

The study group included OC patients who underwent excision of the primary ovarian tumor and BM between 2000 and 2015. The study material included archived, anonymized, formalin-fixed, paraffin-embedded samples (FFPE) of BMs, primary ovarian tumors, and normal tissues (ovary, fallopian tube, uterine corpus, uterine cervix in one, two, four, and three cases respectively). Demographic, clinicopathologic, and clinical follow-up data were extracted from medical records. All data were coded to ensure the full protection of personal information; therefore, patient consent was not sought. Informed consent was waived and approved by the Ethics Committee of the Military Institute of Medicine in Warsaw, Poland (NR 52/WIM/2015). All experiments were performed in accordance with relevant guidelines and regulations.

### DNA extraction

The genomic analysis encompassed 30 FFPE samples retrospectively collected from a cohort of ten OC patients who developed BM. The pathologic diagnosis was independently confirmed by two board-certified pathologists (RP and MB) who reviewed FFPE tissue sections stained with hematoxylin and eosin. The DNA was extracted from paraffin blocks using QIAamp® DNA FFPE Tissue Kit (Qiagen) according to the manufacturer’s instructions, with minor modifications: paraffin removal with xylene was repeated twice, incubation at 56 °C was performed overnight, and an additional dose of Proteinase K (20 μl of > 600 mAU/mLsolution) was added after three hours of incubation. The final elution volume was 40 μl. After extraction, DNA concentration and purity were determined with PicoGreenTM (Thermofisher Scientific) using a Victor3TMPlate Reader (PerkinElmer).

### Sequencing

Whole-exome sequencing was performed using a SureSelectXT Library Prep Kit (2 × 150 bp), with the goal of 100 × coverage for healthy tissue and 200, x coverage for matched BMs and primary tumors. Prepared libraries were sequenced at Macrogen Inc (Seoul, South Korea) using NovaSeq platform (Illumina, Inc). Reads trimming was performed using Trimmomatic. To generate quality control reports for the raw reads, we used GATK 3.8 and Picard 2.17.0. The sequencing coverage for metastasis was in the range of 23–113x; 34–111 × for primary tumors and 21–58 × for controls.

### Sequence alignment and variant calling

Sequence read alignment was performed using the Burrows Wheeler Alignment algorithm (BWA-MEM, version 0.7.17). Somatic variant calling was performed using VarDict (vardict-java 1.7.0) and Mutect2 (GATK 3.8) for each primary tumor versus corresponding normal tissue and BMs versus corresponding normal tissue. In addition to somatic variant calling, germline calls were generated for all normal tissue samples using Freebayes (1.1.0.46) in joint (cohort-based) calling mode. In both cases, variants were annotated with over 90 variant annotation features using snpEff (4.3.1t) and Gemini (0.30.2). Variant annotations included protein-coding effects, population allele frequencies (from the 1000 Genomes Project, ESP, ExAC, and gnomAD), prior observations in cancer genomes (including TCGA and COSMIC v71), and other features.

### Variant filtering

Starting from the initial calls, a set of high-confidence somatic variant calls was derived using the following filtering criteria: (i) the variant corresponds to a high-quality call (PASS), (ii) the variant is not in the CSE or GRC low-complexity regions (in which variant callers typically have a high false-positive rate), (iii) the variant has a low frequency in germline population cohorts (either absent or maximal observed frequency in all annotated population cohorts; < 0.0001, corresponding to 0.01%), and (iv) the variant is either classified as impactful on protein sequence (HIGH or MEDIUM using Gemini severity criteria) or has been observed previously in cancer cohorts (TCGA or COSMIC) more than once. For germline variants, similar criteria were used, with the following modifications: (i) the maximal germline variant frequency was 0.01 (1%), (ii) in addition to previously observed in cancer cohorts (TCGA or COSMIC) occurred more than once, and (iii) were earlier reported in OMIM or ClinVar.

### Functional pathway-level analysis

The functional enrichment of somatic variants was computed using the filtered high-confidence call set (filtering criteria specified above), further limiting inclusion to the variants called by VarDict and Mutect2. Enrichment significance (p-value, FDR) was computed using the Sample-population Level Analysis of Pathway Alterations Enrichments (SLAPenrich) algorithm over the REACT and KEGG pathway collections, as well as the GO-BP^17,18,53^. Enrichment was computed by considering two types of somatic mutation profiles: (i) genes mutated in primary tumors versus the corresponding normal tissue and (ii) genes mutated in BMs versus the corresponding primary tumors. Considering the significant overlap between various pathways and the role of DNA repair deficits in OC, resulting in numerous random mutations, the potential disturbance of some pathways may be incidental despite the high enrichment scores and low FDR values. To increase the chance of detecting accurate mechanisms that are crucial to BM, we employed a set of assumptions: a high enrichment score (FDR < 0.01), relatively universal pathway disturbance (mutations in at least half of cases), specific pathways (expected number of mutations < 3), and detection across collections (at least half of mutations in common).

## Supplementary Information

Supplementary Information.

## Data Availability

Results described in the publication are based on WES data which includes sensitive information in the form of patient specific germline variants. The datasets generated and/or analysed during the current study are available in the Military Institute of Medicine repository, (https://repozytorium.wim.mil.pl). Access to raw data can be provided following contact with corresponding author.

## References

[1] Sung H (2021). Global cancer statistics 2020: GLOBOCAN estimates of incidence and mortality worldwide for 36 cancers in 185 countries. CA Cancer J. Clin..

[2] Hennessy BT, Coleman RL, Markman M (2009). Ovarian cancer. Lancet.

[3] Kumar L (2003). Central nervous system metastases from primary epithelial ovarian cancer. Cancer Control.

[4] Cohen ZR (2004). Brain metastases in patients with ovarian carcinoma: Prognostic factors and outcome. J. Neuro-Oncol..

[5] Bowtell DD (2015). Rethinking ovarian cancer II: reducing mortality from high-grade serous ovarian cancer. Nat. Rev. Cancer.

[6] The Cancer Genome Atlas Research Network (2011). Integrated genomic analyses of ovarian carcinoma. Nature.

[7] The Cancer Genome Atlas Network (2012). Comprehensive molecular portraits of human breast tumours. Nature.

[8] Balendran S (2017). Next-Generation Sequencing-based genomic profiling of brain metastases of primary ovarian cancer identifies high number of BRCA-mutations. J. NeuroOncol..

[9] Szarszewska M (2019). Significance of BRCA1 expression in breast and ovarian cancer patients with brain metastasis—A multicentre study. Adv. Med. Sci..

[10] Sekine M (2013). Increased incidence of brain metastases in BRCA1-related ovarian cancers. J. Obstet. Gynaecol. Res..

[11] Mittica G (2017). Androgen receptor status predicts development of brain metastases in ovarian cancers. Oncotarget.

[12] Lancaster JM, Dressman HK, Clarke JP, Sayer RA, Martino MA, Cragun JM (2006). Identification of genes associated with ovarian cancer metastasis using microarray expression analysis. Int. J. Gynecol. Cancer.

[13] Matsuo K (2011). Multidrug resistance gene (MDR-1) and risk of brain metastasis in epithelial ovarian, fallopian tube, and peritoneal cancer. Am. J. Clin. Oncol..

[14] Brodsky AS (2014). Expression profiling of primary and metastatic ovarian tumors reveals differences indicative of aggressive disease. PLoS ONE.

[15] Bitler BG (2011). Wnt5a suppresses epithelial ovarian cancer by promoting cellular senescence. Cancer Res..

[16] Burkhalter RJ (2011). Integrin regulation of beta-catenin signaling in ovarian carcinoma. J. Biol. Chem..

[17] Gaffney SG, Townsend JP (2016). PathScore: a web tool for identifying altered pathways in cancer data. Bioinformatics.

[18] Griss J (2020). ReactomeGSA: Efficient multi-omics comparative pathway analysis. Mol. Cell Proteomics.

[19] Chatzinikolaou G, Karakasilioti I, Garinis GA (2014). DNA damage and innate immunity: Links and trade-offs. Trends Immunol..

[20] Mouw KW, Goldberg MS, Konstantinopoulos PA, D'Andrea AD (2017). DNA damage and repair biomarkers of immunotherapy response. Cancer Discov..

[21] Turner NC, Reis-Filho JS (2012). Genetic heterogeneity and cancer drug resistance. Lancet Oncol..

[22] Brastianos PK (2015). Genomic characterization of brain metastases reveals branched evolution and potential therapeutic targets. Cancer Discov..

[23] Hwang WT, Adams SF, Tahirovic E, Hagemann IS, Coukos G (2012). Prognostic significance of tumor-infiltrating T cells in ovarian cancer: A meta-analysis. Gynecol. Oncol..

[24] Sato E (2005). Intraepithelial CD8+ tumor-infiltrating lymphocytes and a high CD8+/regulatory T cell ratio are associated with favorable prognosis in ovarian cancer. Proc. Natl. Acad. Sci. USA.

[25] Kumar V (2019). Toll-like receptors in the pathogenesis of neuroinflammation. J. Neuroimmunol..

[26] Choi YJ (2019). Integrative immunologic and genomic characterization of brain metastasis from ovarian/peritoneal cancer. Pathol. Res. Pract..

[27] Hamanishi J (2015). Safety and antitumor activity of anti-PD-1 antibody, nivolumab, in patients with platinum-resistant ovarian cancer. J. Clin. Oncol..

[28] Varga A (2019). Pembrolizumab in patients with programmed death ligand 1-positive advanced ovarian cancer: Analysis of KEYNOTE-028. Gynecol. Oncol..

[29] Kienast Y (2010). Real-time imaging reveals the single steps of brain metastasis formation. Nat. Med..

[30] Sevenich L (2014). Analysis of tumour- and stroma-supplied proteolytic networks reveals a brain-metastasis-promoting role for cathepsin *S*. Nat. Cell Biol..

[31] Jakoš T, Pišlar A, Jewett A, Kos J (2019). Cysteine cathepsins in tumor-associated immune cells. Front. Immunol..

[32] De Luca C, Colangelo AM, Virtuoso A, Alberghina L, Papa M (2020). Neurons, glia, extracellular matrix and neurovascular unit: A systems biology approach to the complexity of synaptic plasticity in health and disease. Int. J. Mol. Sci..

[33] Chevilley A (2015). Impacts of tissue-type plasminogen activator (tPA) on neuronal survival. Front. Cell Neurosci..

[34] Huang J (2021). Extracellular matrix and its therapeutic potential for cancer treatment. Signal Transduct. Target. Ther..

[35] Korade Z, Kenworthy AK (2008). Lipid rafts, cholesterol, and the brain. Neuropharmacology.

[36] Tracey TJ, Steyn FJ, Wolvetang EJ, Ngo ST (2018). Neuronal lipid metabolism: multiple pathways driving functional outcomes in health and disease. Front. Mol. Neurosci..

[37] Olsen ASB, Færgeman NJ (2017). Sphingolipids: membrane microdomains in brain development, function and neurological diseases. Open Biol..

[38] Tiwary S (2018). Metastatic brain tumors disrupt the blood-brain barrier and alter lipid metabolism by inhibiting expression of the endothelial cell fatty acid transporter Mfsd2a. Sci. Rep..

[39] Kwok ZH, Wang C, Jin Y (2021). Extracellular vesicle transportation and uptake by recipient cells: A critical process to regulate human diseases. Processes (Basel).

[40] Escrevente C, Keller S, Altevogt P, Costa J (2011). Interaction and uptake of exosomes by ovarian cancer cells. BMC Cancer.

[41] Yue KY (2019). Neurons can upregulate Cav-1 to increase intake of endothelial cells-derived extracellular vesicles that attenuate apoptosis via miR-1290. Cell Death Dis..

[42] Liu J (2021). The role of the Golgi apparatus in disease (review). Int. J. Mol. Med..

[43] de Aguiar Pastore Silva J, Emiliade Souza Fabre M, Waitzberg DL (2015). Omega-3 supplements for patients in chemotherapy and/or radiotherapy: A systematic review. Clin. Nutr..

[44] Dorsam RT, Gutkind JS (2007). G-protein-coupled receptors and cancer. Nat. Rev. Cancer.

[45] Choi JW (2010). LPA receptors: Subtypes and biological actions. Annu. Rev. Pharmacol. Toxicol..

[46] Lee Z (2006). Lysophosphatidic acid is a major regulator of growth-regulated oncogene alpha in ovarian cancer. Cancer Res..

[47] Wang W (2020). Lysophosphatidic acid induces tumor necrosis factor-alpha to regulate a pro-inflammatory cytokine network in ovarian cancer. FASEB J..

[48] de Amaral RF (2021). Microglial lysophosphatidic acid promotes glioblastoma proliferation and migration via LPA1 receptor. J. Neurochem..

[49] Ranzani M (2017). Revisiting olfactory receptors as putative drivers of cancer. Wellcome Open Res..

[50] Maßberg D, Hatt H (2018). Human olfactory receptors: Novel cellular functions outside of the nose. Physiol. Rev..

[51] Graf RP (2021). Association of copy number variation signature and survival in patients with serous ovarian cancer. JAMA Netw. Open.

[52] Norquist BM (2016). Inherited mutations in women with ovarian carcinoma. JAMA Oncol..

[53] Kanehisa M, Goto S (2000). KEGG: Kyoto encyclopedia of genes and genomes. Nucleic Acids Res..

