# Supplementary material for: Pathway-level mutation analysis in primary high-grade serous ovarian cancer and matched brain metastases
Source: Sci Rep. 2022 Nov 29;12:20537. doi: 10.1038/s41598-022-23788-4 (PMC9708673; doi:10.1038/s41598-022-23788-4)
Supplement: Supplementary file 1 — Supplementary Information. [file 41598_2022_23788_MOESM1_ESM.docx]

**Pathway-level mutation analysis in primary high-grade serous ovarian cancer
and matched brain metastases**

**Duchnowska, R. et al.**

**Supplementary Figure 1 and Table 1.**

**Fig. S1. Oncoprint for the most frequently mutated genes.**

Oncoprint showing the most frequent mutations in cancer-related genes observed in the study group. Germline and somatic mutations within each gene are specifically depicted. The plot was generated using R statistical software version 4.1.2 [R Core Team (2021). R: A language and environment for statistical computing. R Foundation for Statistical Computing, Vienna, Austria. URL [https://www.R-project.org/](https://www.r-project.org/)] with ComplexHeatmap package version 2.10.0 [Gu Z, Eils R, Schlesner M (2016). „Complex heatmaps reveal patterns and correlations in multidimensional genomic data“ Bioinformatics, URL https://academic.oup.com/bioinformatics/article/32/18/2847/1743594].


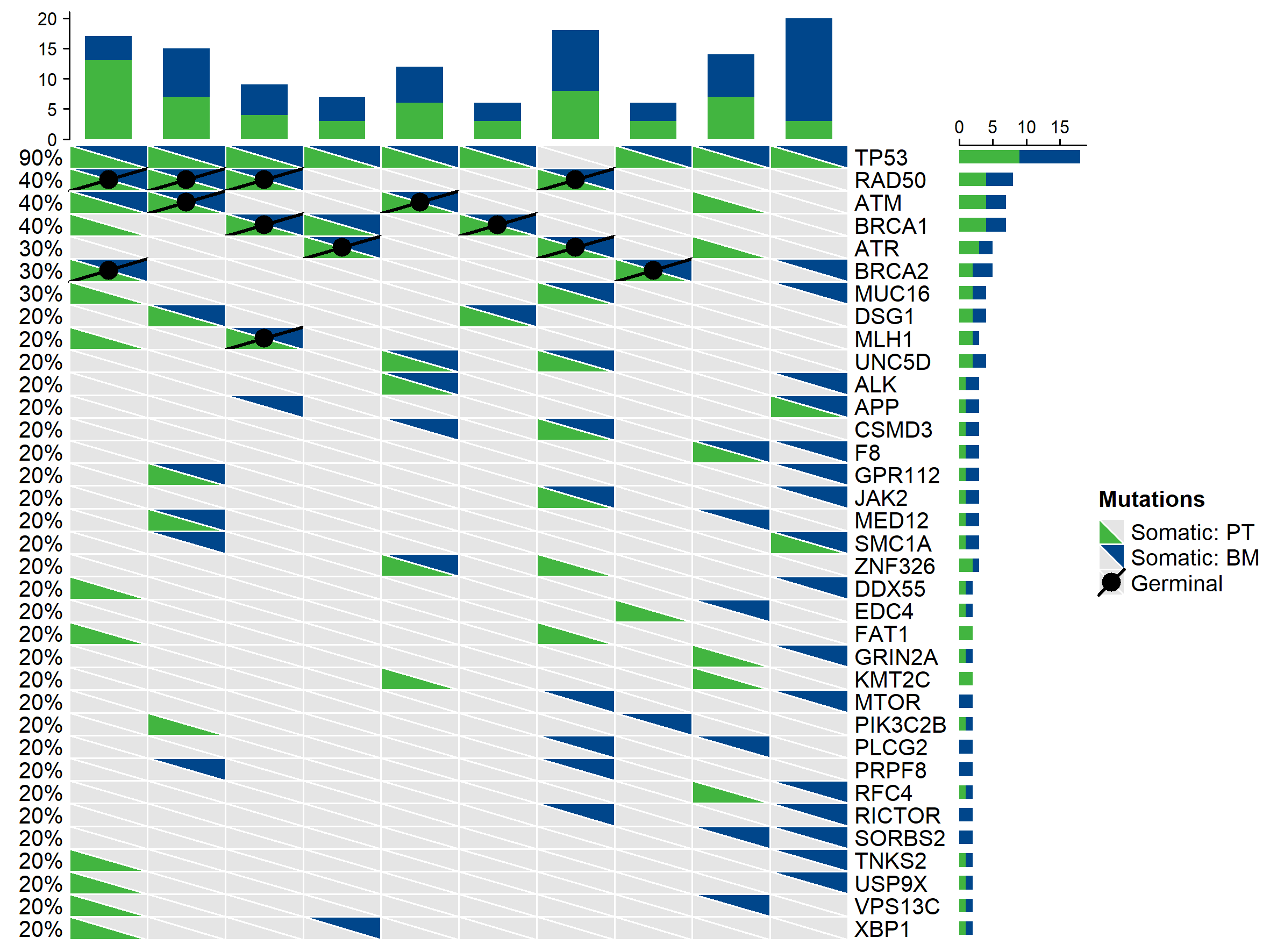


Table S1. Main pathway-level differences between BMs and primary OC using REACT, KEGG, and GO-BP pathway collections.

| **Pathway ID** | **Pathway Label** | **P value** | **FDR** | **Pathway ID** | **Pathway Label** | **P value** | **FDR** |
| --- | --- | --- | --- | --- | --- | --- | --- |
| REACT_118572 | Degradation of the extracellular matrix | 6.28E-05 | 0.004802197 | REACT_160158 | Role of phospholipids in phagocytosis | 0.00027 | 0.008373369 |
| GO-BP: GO:0022411 | cellular component disassembly | 0.001463 | 0.004376546 | REACT_160123 | Fcgamma receptor (FCGR) dependent phagocytosis | 1.89E-05 | 0.004802197 |
| GO-BP:GO:0030198 | extracellular matrix organization | 4.17E-05 | 0.000493273 | REACT_160086 | Regulation of actin dynamics for phagocytic cup formation | 0.000296 | 0.008373369 |
| GO-BP:GO:0043062 | extracellular structure organization | 4.17E-05 | 0.000493273 | REACT_6966 | Toll-Like Receptors Cascades | 4.45E-05 | 0.004802197 |
| GO-BP:GO:0022617 | extracellular matrix disassembly | 4.17E-05 | 0.000493276 | REACT_6894 | Toll Like Receptor 4 (TLR4) Cascade | 0.000282 | 0.008373369 |
| REACT_20676 | Cell junction organization | 0.000112 | 0.005989953 | REACT_6890 | Activated TLR4 signalling | 0.000147 | 0.005989953 |
| REACT_111155 | Cell-Cell communication | 0.000245 | 0.008373369 | REACT_25281 | TRIF-mediated TLR3/TLR4 signaling | 0.000147 | 0.005989953 |
| GO-BP:GO:0045216 | cell-cell junction organization | 5.77E-07 | 5.62255E-05 | REACT_6809 | MyD88-independent TLR3/TLR4 cascade | 0.000147 | 0.005989953 |
| GO-BP:GO:0034332 | adherens junction organization | 5.62E-06 | 0.000187317 | REACT_6783 | Toll Like Receptor 3 (TLR3) Cascade | 0.000147 | 0.005989953 |
| GO-BP:GO:0034330 | cell junction organization | 1.65E-06 | 9.51823E-05 | KEGG:hsa04620 | Toll-like receptor signaling pathway | 0.000928 | 0.008119230 |
| GO-BP:GO:0034329 | cell junction assembly | 7.55E-07 | 6.64656E-05 | KEGG:hsa04142 | Lysosome | 0.000402 | 0.005984726 |
| GO-BP:GO:0032956 | regulation of actin cytoskeleton organization | 3.06E-05 | 0.000410176 | KEGG:hsa04145 | Phagosome | 0.000967 | 0.008119230 |
| GO-BP:GO:0032970 | regulation of actin filament-based process | 5.22E-05 | 0.000549499 | KEGG:hsa04217 | Necroptosis | 0.000228 | 0.004195038 |
| GO-BP:GO:1903827 | regulation of cellular protein localization | 4.96E-05 | 0.000532442 | GO-BP:GO:0001819 | positive regulation of cytokine production | 4.46E-05 | 0.000500395 |
| KEGG:hsa04110 | Cell cycle | 0.001024 | 0.008126613 | GO-BP:GO:0045089 | positive regulation of innate immune response | 0.000403 | 0.001822607 |
| GO-BP:GO:0007346 | regulation of mitotic cell cycle | 1.77E-05 | 0.000323030 | GO-BP:GO:0002218 | activation of innate immune response | 0.000285 | 0.001470222 |
| GO-BP:GO:0045786 | negative regulation of cell cycle | 0.000672 | 0.002591549 | GO-BP:GO:0006909 | phagocytosis | 1.25E-05 | 0.000271483 |
| GO-BP:GO:0010948 | negative regulation of cell cycle process | 4.4E-05 | 0.000499655 | GO-BP:GO:0002252 | immune effector process | 3.05E-05 | 0.000410176 |
| GO-BP:GO:1901987 | regulation of cell cycle phase transition | 0.000904 | 0.003154745 | GO-BP:GO:0045321 | leukocyte activation | 0.000669 | 0.002587660 |
| GO-BP:GO:1901990 | regulation of mitotic cell cycle phase transition | 0.000607 | 0.002439200 | GO-BP:GO:0001817 | regulation of cytokine production | 5.27E-06 | 0.000181894 |
| GO-BP:GO:0045861 | negative regulation of proteolysis | 0.000351 | 0.001687283 | GO-BP:GO:0031349 | positive regulation of defense response | 0.001811 | 0.005113302 |
| GO-BP:GO:0051302 | regulation of cell division | 7.56E-05 | 0.000683566 | GO-BP:GO:0045088 | regulation of innate immune response | 0.000643 | 0.002537200 |
| GO-BP:GO:0045930 | negative regulation of mitotic cell cycle | 0.0034 | 0.007830233 | GO-BP:GO:0002764 | immune response-regulating signaling pathway | 0.000262 | 0.001392612 |
| GO-BP:GO:0007093 | mitotic cell cycle checkpoint | 0.001135 | 0.003695701 | GO-BP:GO:0002757 | immune response-activating signal transduction | 6.27E-05 | 0.000596932 |
| GO-BP:GO:0051783 | regulation of nuclear division | 7.85E-05 | 0.000700607 | GO-BP:GO:0002253 | activation of immune response | 9.66E-05 | 0.000785113 |
| GO-BP:GO:0007088 | regulation of mitotic nuclear division | 3.23E-05 | 0.000419047 | GO-BP:GO:0050778 | positive regulation of immune response | 7.62E-06 | 0.000216988 |
| KEGG:Nucleotide metabolism | Nucleotide metabolism | 4.08E-05 | 0.003147645 | GO-BP:GO:0050776 | regulation of immune response | 0.000117 | 0.000886518 |
| KEGG:hsa00230 | Purine metabolism | 4.08E-05 | 0.003147645 | GO-BP:GO:0002221 | pattern recognition receptor signaling pathway | 1.03E-05 | 0.000255091 |
| GO-BP:GO:0055086 | nucleobase-containing small molecule metabolic process | 2.41E-07 | 2.93529E-05 | GO-BP:GO:0002224 | toll-like receptor signaling pathway | 3.94E-06 | 0.000156039 |
| GO-BP:GO:0006753 | nucleoside phosphate metabolic process | 2.87E-08 | 9.3999E-06 | GO-BP:GO:0002758 | innate immune response-activating signal transduction | 0.000211 | 0.001217225 |
| GO-BP:GO:0009117 | nucleotide metabolic process | 2.87E-08 | 9.3999E-06 | REACT_19323 | Sphingolipid metabolism | 0.000293 | 0.008373369 |
| GO-BP:GO:0072521 | purine-containing compound metabolic process | 1.68E-08 | 7.29297E-06 | GO-BP:GO:0006643 | membrane lipid metabolic process | 5.37E-05 | 0.000557463 |
| GO-BP:GO:0006163 | purine nucleotide metabolic process | 6.64E-09 | 5.18276E-06 | GO-BP:GO:0006665 | sphingolipid metabolic process | 1.81E-05 | 0.000328301 |
| GO-BP:GO:0019693 | ribose phosphate metabolic process | 3.01E-09 | 2.96663E-06 | GO-BP:GO:1903509 | liposaccharide metabolic process | 8.96E-06 | 0.000241626 |
| GO-BP:GO:0009150 | purine ribonucleotide metabolic process | 3.01E-09 | 2.96663E-06 | GO-BP:GO:0006664 | glycolipid metabolic process | 8.96E-06 | 0.000241626 |
| GO-BP:GO:0009259 | ribonucleotide metabolic process | 3.01E-09 | 2.96663E-06 | REACT_14828 | Class A/1 (Rhodopsin-like receptors) | 0.000381 | 0.009403258 |
| GO-BP:GO:0009187 | cyclic nucleotide metabolic process | 6.71E-06 | 0.000205014 | REACT_21340 | GPCR ligand binding | 9.78E-06 | 0.004802197 |
| GO-BP:GO:0072522 | purine-containing compound biosynthetic process | 2.55E-06 | 0.000120058 | KEGG:hsa04080 | Neuroactive ligand-receptor interaction | 0.001369 | 0.009148565 |
| REACT_121175 | PI Metabolism | 0.000205 | 0.007524250 | GO-BP:GO:0007186 | G-protein coupled receptor signaling pathway | 3.12E-05 | 0.000412281 |
| REACT_121025 | Synthesis of PIPs at the plasma membrane | 0.000104 | 0.005989953 | REACT_11235 | trans-Golgi Network Vesicle Budding | 6.28E-05 | 0.004802197 |
| REACT_120870 | Phospholipid metabolism | 5.15E-05 | 0.004802197 | REACT_19187 | Clathrin derived vesicle budding | 6.28E-05 | 0.004802197 |
| REACT_18283 | G alpha (q) signalling events | 0.000376 | 0.009403258 | REACT_19287 | Lysosome Vesicle Biogenesis | 2.26E-05 | 0.004802197 |
| KEGG:hsa04070 | Phosphatidylinositol signaling system | 0.000513 | 0.006556776 | GO-BP:GO:0006892 | post-Golgi vesicle-mediated transport | 0.000623 | 0.002483417 |
| KEGG:hsa00562 | Inositol phosphate metabolism | 4.42E-05 | 0.003147645 | GO-BP:GO:0048193 | Golgi vesicle transport | 3.95E-05 | 0.000473696 |
| GO-BP:GO:0006644 | phospholipid metabolic process | 7.81E-05 | 0.000698378 | REACT_15488 | Olfactory Signaling Pathway | 3.76E-05 | 0.004802197 |
| GO-BP:GO:0006650 | glycerophospholipid metabolic process | 6.14E-05 | 0.000591707 | KEGG:hsa04740 | Olfactory transduction | 0.000558 | 0.006633076 |
| GO-BP:GO:0046486 | glycerolipid metabolic process | 7.37E-06 | 0.000213085 | KEGG:hsa04742 | Taste transduction | 1.08E-05 | 0.003147645 |
| GO-BP:GO:0045017 | glycerolipid biosynthetic process | 9.27E-05 | 0.000242707 | GO-BP:GO:0007186 | G-protein coupled receptor signaling pathway | 3.12E-05 | 0.000412281 |
| GO-BP:GO:0046474 | glycerophospholipid biosynthetic process | 6.4E-06 | 0.000204514 | GO-BP:GO:0007606 | sensory perception of chemical stimulus | 1.7E-07 | 2.20646E-05 |
| GO-BP:GO:0008654 | phospholipid biosynthetic process | 6.4E-06 | 0.000204514 | GO-BP:GO:0050906 | detection of stimulus involved in sensory perception | 2.14E-05 | 0.000363131 |
| GO-BP:GO:0008610 | lipid biosynthetic process | 1.61E-07 | 2.20646E-05 | GO-BP:GO:0009593 | detection of chemical stimulus | 2E-07 | 2.52216E-05 |
| GO-BP:GO:0090407 | organophosphate biosynthetic process | 7.86E-08 | 1.34498E-05 | GO-BP:GO:0050907 | detection of chemical stimulus involved in sensory perception | 5.56E-08 | 1.31559E-05 |
| GO-BP:GO:0046488 | phosphatidylinositol metabolic process | 1.51E-05 | 0.000290439 | GO-BP:GO:0007608 | sensory perception of smell | 2.26E-06 | 0.000114424 |
| REACT_160158 | Role of phospholipids in phagocytosis | 0.00027 | 0.008373369 | GO-BP:GO:0050911 | detection of chemical stimulus involved in sensory perception of smell | 1.14E-06 | 8.07639E-05 |

REACT: Reactome; KEGG: Kyoto Encyclopedia of Genes and Genomes, GO-BP: Gene Ontology Biological Process
